# Effects of mobile Health (mHealth) application on cervical cancer prevention knowledge and screening among women social support groups with low-socioeconomic status in Mysuru city, Southern India

**DOI:** 10.1371/journal.pone.0273070

**Published:** 2022-09-01

**Authors:** Chandana Hombaiah, B. Madhu, Arun Gopi, M. R. Narayana Murthy

**Affiliations:** Department of Community Medicine, JSS Medical College, JSS Academy of Higher Education & Research, Mysuru, Karnataka, India; Ordu University, TURKEY

## Abstract

**Background:**

Cervical cancer is an important area of action because of the mortality and morbidity of the disease, and the potential for effective prevention by screening. Involving the social support groups by health education improves cervical cancer screening and early detection of the disease in the community. In the ongoing efforts to strengthen cervical cancer prevention, control, and management, digital health and technology will have a significant role to play.

**Objective:**

To assess the effectiveness of the mHealth-based intervention on cervical cancer preparedness among women social support groups.

**Materials & methods:**

A pre-post interventional study was conducted among women social support groups from lower socio-economic status, identified from the field practice area. Purposive sampling technique was employed. A Cervical Cancer Awareness Measure (CAM) instrument was used to assess the cancer preparedness among the social support group women After taking inputs from the stakeholders’ mobile health application was developed. The mHealth educational intervention was given to 102 women. Both pre-and post-test questionnaires were administered through mHealth application to assess the change in knowledge after a gap of 1 month to 2 months. The data obtained was coded and entered into Microsoft Excel worksheet 2016 and was later imported and analyzed using SPSS version 22 (licensed to the Institute). The difference in median scores of knowledge and practice were interpreted as statistically significant at p value of < 0.05.

**Results & conclusion:**

Before the intervention only 13 (12.7%) of them had heard about cervical cancer. There was a significant increase in the knowledge about warning signs & symptoms, risk factors of cervical cancer, and HPV vaccination. Around 5% increase in Pap smear test uptake.

## Introduction

Cervical cancer is an important area of action because of the mortality and morbidity of the disease, and the potential for effective prevention by screening. It is the fourth most common cancer among women globally and one of the leading cancer causing death in developing countries [1]. As per the Indian Human Papilloma Virus Information Centre 2019 report, 96,922 cases and 60,078 deaths occur annually due to cervical cancer [2] and the crude incidence rate for cervical cancer per year is 14.9/1,00,000 [2]. According to a Hospital-based Cancer Registry from Bengaluru, cervical cancer was the leading site, accounting for around 29.4% of female cancers followed by breast (14.2%) [3]. Among the general population, low-socio-economic status women are at high risk of increased incidence and mortality due to cervical cancer [4, 5].

Health education in the prevention of cancer will help in acquiring health-promoting knowledge, which will motivate participants to make the recommended, evidence-based behavioral modifications like screening that would reduce cancer-related morbidity and mortality [6, 7].

Social support groups also referred to as mutual aid, mutual help, or self-help groups are groups of individuals that offer mutual support to each other [8]. Social support groups have been identified to enhance communication with physicians, improve self-reported health, enhance social / role activity, and reduce hospitalization needs. Involving the social support groups by health education improves cervical cancer screening and early detection of the disease in the community [8–10].

In the ongoing efforts to strengthen cervical cancer prevention, control, and management, digital health and technology will have a significant role to play [11]. Governments are expressing interest in mHealth as a supportive strategy for strengthening health systems and achieving the health-related goals in low- and middle-income countries [12]. The most common application of mHealth is the use of mobile devices to educate consumers about preventive healthcare services [13–15]. Hence, the study was taken up to assess the effectiveness of the mHealth-based intervention on cervical cancer prevention knowledge and screening among women social support groups with low-socioeconomic status.

## Materials & methods

### Study tool

A Cervical Cancer Awareness Measure (CAM) instrument was used. The survey instrument (the Cervical CAM) was developed by the University College of London (UCL) Health Behaviour Research Centre, in collaboration with the Department of Health Cancer Team and The Eve Appeal, with funding from The Eve Appeal. It forms part of the Cervical Cancer Awareness and Symptoms Initiative (CCASI). It is based on a generic CAM developed by Cancer Research UK, University College London, King’s College London, and Oxford University in 2007–08 [16]. The questionnaire was modified to the study settings [S1 File]. The questionnaire had items on knowledge about warning signs and symptoms (11 items), knowledge on risk factors about cervical cancer (11 items), knowledge about vaccination, and practice level of Pap smear. The response options for knowledge about warning signs and symptoms and knowledge about vaccination were: Yes, No, and Don’t know (Yes was coded as 2, No was coded as 1, and Don’t know was coded as 0). The minimum score and maximum score for knowledge about warning signs and symptoms were 0 and 22. Since knowledge about vaccination was assessed with 1 question, the minimum score was 0 and the maximum score was 2. Knowledge on risk factors about cervical cancer was assessed using a 5-point Likert scale: 1 for Strongly disagree, 2 for Disagree, 3 for Don’t know, 4 for Agree, and 5 for strongly agree. The minimum score and maximum score for knowledge about risk factors were 11 and 55 respectively. A Score of above 33 was regarded as the participants were aware and a score of 33 and less than 33 as the participants were not aware of risk factors of cervical cancer. Response options for practice level of Pap test were: Yes (coded as 2) and No (coded as 1). Internal consistency was assessed by Cronbach’s alpha. The Cronbach’s alpha for the entire set of questionnaires was 0.739 and the values for each domain as follows: 0.955 for knowledge on warning signs and symptoms, and 0.71 for knowledge on risk factors about cervical cancer.

### Inclusion & exclusion criteria

Lower socioeconomic status women aged between 18–60 years belonging to social support groups from the field practice area of the Medical College were included in the study. Women who were pregnant, lactating, suffering from cervical cancer and bed ridden were excluded from the study.

### Data collection

A pre-post interventional study was conducted among women social support groups from lower socio-economic status, identified from the field practice area of the Medical College from January 2018 to June 2020. Purposive sampling technique was employed. Social support groups are informal groups of people who come together to address their common problems [17]. In India, self-help groups are typically woman-oriented, and most of their activities concentrate on savings and credit activities (apart from other activities aimed at empowering women, health and educational attainment, etc.) [18]. In the field practice area, these groups are called as ‘Stree shakthi sangas’. There were around 24 women social support groups in the area. Each group consisted of about 6–12 women. Two social support groups were selected randomly through chit method. A pilot study was conducted among those two social support groups (14 women). Socio-demographic profiles of these women were collected, which included age, marital status, education, occupation, socio-economic status, and history of cancer among family and friends. Secondly, knowledge about cervical cancer preparedness was assessed by the questionnaire. After taking inputs from the stakeholders’ mobile health application was developed. Mobile health application has an interactive voice response regarding cervical cancer and also it would send a reminder for the women of social support groups regarding screening tests. We invited around 20 social support group women for the study, but only 8 groups could give a date for us, as many of them were not meeting due to the pandemic situation. The educational intervention was given to 102 women from 10 social support groups (this includes pilot study groups). Both pre-and post-test questionnaires were administered through mHealth application to assess the change in knowledge and practice after a gap of 1 month to 2 months. Each group was called and Illiterate women were interviewed for the same and health education module was given to them to listen to the modules with the help of voice notes in the mHealth application.

### Educational intervention- mHealth application

The mobile health application was developed by ZMQ development, Delhi, India. It is available both in English and in the local language i.e., Kannada in writing as well as in the audio format. The application asks for the registration of the candidate first. In the registration process, application asks for name, age, marital status, education, occupation, and monthly income for the house. After registration, a pre-test questionnaire will be displayed and then it will open up the health education modules about cervical cancer. After the modules, the participant is supposed to complete the post-test questionnaire too. Illiterate women were assisted to answer the same. It will show the cumulative percentage of the pre-test and post-test to the participant, for them to know how much their knowledge has been improved. For the study purpose, post-test questionnaire was implemented again among the participants after 1–2 months. Lastly, there is a self-screening tool, which will ask whether the participants have undergone a Pap test or not, if no, then it‘ll give information as to where they can get it done depending on their age (Pap test is not recommended for <21years). If the participant has already got the Pap test, then they can record the date in the application and they are supposed to mention whether the test result was positive or negative. If negative then it will let them know when their next visit to the health care facility to get the Pap test. If positive, then the application shows what are the confirmatory tests they can get done and management for the same [S2 File].

### Ethics

Details about the study were explained to all the women in the social support groups and a written informed consent was taken from all the women who were willing to participate in the study. The study was approved by the Institutional Ethics Committee of JSS AHER (JSS/MC/PG/4623/2018-19).

### Statistical analysis

The data obtained was coded and entered into Microsoft Excel worksheet 2016 and was later imported and analyzed using SPSS version 22 (licensed to JSS AHER). Descriptive statistical measures like percentages and proportions were used to express qualitative data. Quantitative data that were normally distributed were expressed as mean and standard deviation while non-normally distributed data were expressed as median and interquartile range. Data was represented as tables and graphs as relevant. The difference between the median knowledge and practice scores before and after the intervention was assessed using the Wilcoxon Sign Rank test. Kruskal Wallis test was conducted to assess the difference between median knowledge scores of warning signs and symptoms, risk factors of cervical cancer and HPV vaccination, and practice scores of Pap test across the categories of socio-demographic variables like age category, education category, socioeconomic status, and marital status. Mann-Whitney U test was conducted to assess the difference between medians of knowledge scores of warning signs and symptoms, risk factors of cervical cancer and HPV vaccination, and practice scores of Pap test across the occupational status. Differences were interpreted as statistically significant at p < 0.05.

## Results

Among 102 subjects, most i.e., 47 (46.1%) of them belonged to the age group of 18–30 years, 35 (34.3%) of them belonged to 30–45 years, and 20 (19.6%) of them to 45–60 years age group. The mean age of the participants was 35.25 +11.28 years. Around 19 (18.6%) of them were illiterate, 42 (41.2%) of them had finished schooling (1^st^ to 10^th^ Std.), and 41 (40.2%) had received more than 10 years of education. It was found that 75 (73.5%) of them were unemployed, and 27 (26.4%) of them were employed. According to the modified Kuppuswamy classification of socio-economic status [19], 45 (44.1%) of them belonged to the lower middle class, 51 (50%) of them to the upper-lower class, and 6 (5.9%) of them to the lower class. The majority of them, i.e., 93 (91.2%) of them were married, 5 (4.9%) were single, and 4 (3.9%) were widowed. None of the participants gave any history of cancer among family or friends.

Before the intervention 13 (12.7%) of them had heard about cervical cancer. Comparison of pre-and post-test responses regarding knowledge on warning signs and symptoms of cervical cancer revealed that the majority of them didn’t know about the signs and symptoms of cervical cancer before mHealth educational intervention (**Table 1**). After mHealth educational intervention, the majority of them knew about the signs and symptoms of cervical cancer (**Table 1).**

**Table 1:** Comparison of responses from participants before and after intervention regarding warning signs and symptoms of cervical cancer (n = 102).

Questions regarding knowledge about warning signs and symptoms of cervical cancer	Pre-test (%)			Post-test (%)		
	Yes	No	Don’t know	Yes	No	Don’t know
a. Do you think vaginal bleeding between periods could be a sign of cervical cancer?	5 (4.9)	2 (2)	**95 (93.1)**	**61 (59.8)**	27 (26.5)	14 (13.7)
b. Do you think persistent low back pain could be a sign of cervical cancer?	0	8 (7.8)	**94 (92.2)**	**45 (44.1)**	37 (36.3)	20 (19.6)
c. Do you think a persistent vaginal discharge that smells unpleasant could be a sign of cervical cancer?	9 (8.8)	2 (2)	**91 (89.2)**	**62 (60.8)**	18 (17.6)	22 (21.6)
d. Do you think discomfort or pain during sex could be a sign of cervical cancer?	3 (2.94)	3 (2.94)	**96 (94.12)**	**52 (51)**	35 (34.3)	15 (14.7)
e. Do you think menstrual periods that are heavier or longer than usual could be a sign of cervical cancer?	5 (4.9)	3 (2.9)	**94 (92.2)**	**57 (55.9)**	26 (25.5)	19 (18.6)
f. Do you think persistent diarrhea could be a sign of cervical cancer?	2 (2)	7 (6.8)	**93 (91.2)**	**58 (56.8)**	27 (26.5)	17 (16.7)
g. Do you think vaginal bleeding after menopause could be a sign of cervical cancer?	6 (5.9)	4 (3.9)	**92 (90.2)**	**62 (60.8)**	20 (19.6)	20 (19.6)
h. Do you think persistent pelvic pain could be a sign of cervical cancer?	0	6 (5.9)	**96 (94.1)**	**48 (47.1)**	36 (35.3)	18 (17.6)
i. Do you think vaginal bleeding during or after sex could be a sign of cervical cancer?	2 (2)	3 (2.9)	**97 (95.1)**	**53 (51.9)**	33 (32.4)	16 (15.7)
j. Do you think blood in urine or stool could be a sign of cervical cancer?	2 (2)	3 (2.9)	**97 (95.1)**	**51 (50)**	32 (31.4)	19 (18.6)
k. Do you think unexplained weight loss could be a sign of cervical cancer?	6 (5.9)	2 (2)	**94 (92.1)**	**65 (63.7)**	23 (22.6)	14 (13.7)

Comparison of pre-and post-test responses regarding knowledge on risk factors of cervical cancer has been depicted in **Table 2.**

**Table 2:** Comparison of the pre-and post-test responses regarding knowledge on risk factors of cervical cancer (n = 102).

Variables	*Pre-test (%)*					*Post-test (%)*				
	*Strongly agree*	*Agree*	*Don’t know*	*Disagree*	*Strongly disagree*	*Strongly agree*	*Agree*	*Don’t know*	*Disagree*	*Strongly disagree*
a. Infection with HPV (human papilloma virus)	0	0	**98 (96.1)**	4 (3.9)	0	**4 (3.9)**	**35 (34.3)**	44 (43.2)	11 (10.8)	8 (7.8)
b. Smoking cigarettes	2 (2)	8 (7.8)	**92 (90.2)**	0	0	**17 (16.7)**	**47 (46.1)**	19 (18.6)	11 (10.8)	8 (7.8)
c. Having a weakened immune system (e.g. because of HIV/AIDS, immunosuppressant drugs or having a transplant)	0	3 (2.94)	**96 (94.12)**	3 (2.94)	0	**3 (2.94)**	**45 (44.12)**	35 (34.32)	11 (10.78)	8 (7.84)
d. Long term use of the contraceptive pill	0	0	**98 (96.1)**	4 (3.9)	0	**10 (9.8)**	**50 (49)**	24 (23.5)	12 (11.8)	6 (5.9)
e. Infection with Chlamydia (a sexually transmitted infection)	0	0	**97 (95.1)**	4 (3.9)	1 (1)	**1 (1)**	**27 (26.5)**	47 (46.1)	17 (16.6)	10 (9.8)
f. Having a sexual partner who is not circumcised	0	0	**96 (94.1)**	5 (4.9)	1 (1)	**1 (1)**	**27 (26.5)**	47 (46.1)	14 (13.7)	13 (12.7)
g. Starting to have sex at a young age (before age 17)	0	1 (1)	**98 (96.1)**	3 (2.9)	0	**14 (13.7)**	**49 (48)**	18 (17.7)	11 (10.8)	10 (9.8)
h. Having many sexual partners	0	5 (4.9)	**94 (92.2)**	3 (2.9)	0	**12 (11.7)**	**53 (52)**	21 (20.6)	9 (8.8)	7 (6.9)
i. Having many children	0	1 (1)	**97 (95.1)**	4 (3.9)	0	**2 (2)**	**53 (52)**	28 (27.4)	12 (11.7)	7 (6.9)
j. Having a sexual partner with many previous partners	0	3 (2.94)	**95 (93.14)**	4 (3.92)	0	**3 (2.9)**	**52 (51)**	30 (29.4)	11 (10.8)	6 (5.9)
k. Not going for regular smear (Pap) tests	0	1 (1)	**98 (96.1)**	3 (2.9)	0	**2 (2)**	**50 (49)**	31 (30.4)	12 (11.7)	7 (6.9)

**Fig 1:**
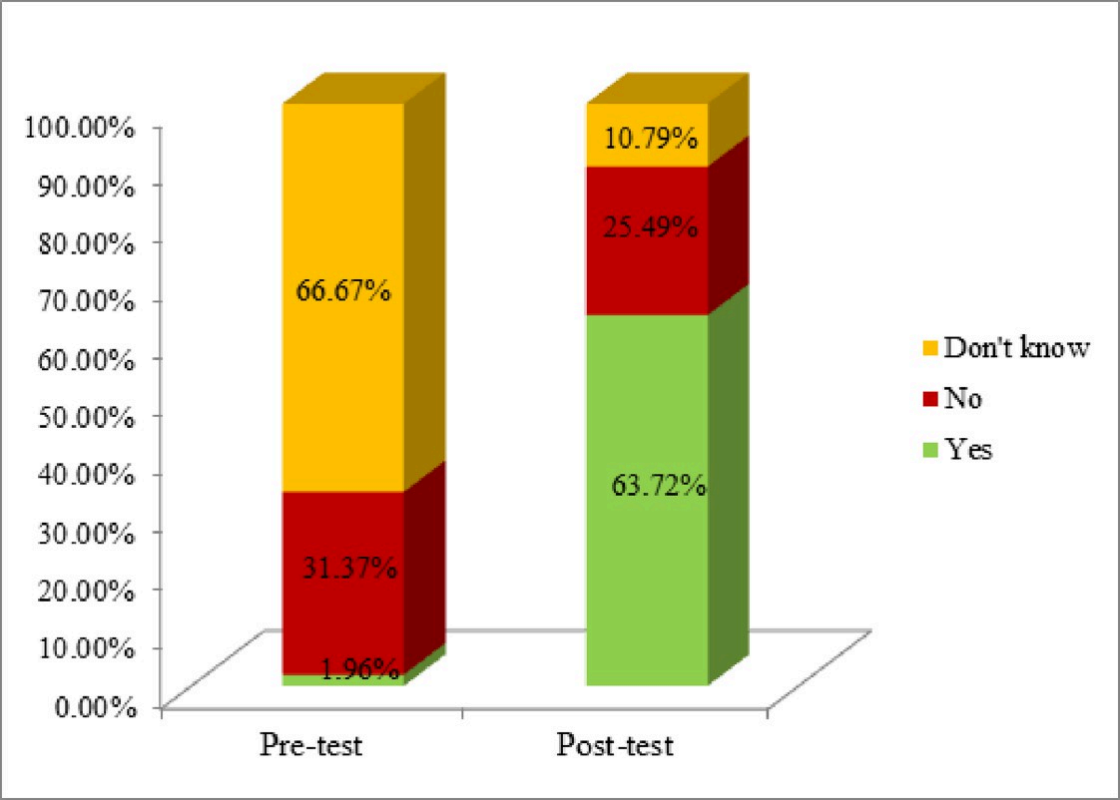
Comparison of participants based on their knowledge about HPV vaccination before and after the intervention (n = 102).

In the current study, before mHealth intervention 68 (66.7%) of them were not aware of HPV Vaccination, but after the intervention, 65 (63.7%) were aware of vaccination (**Fig 1**).

**Fig 2:**
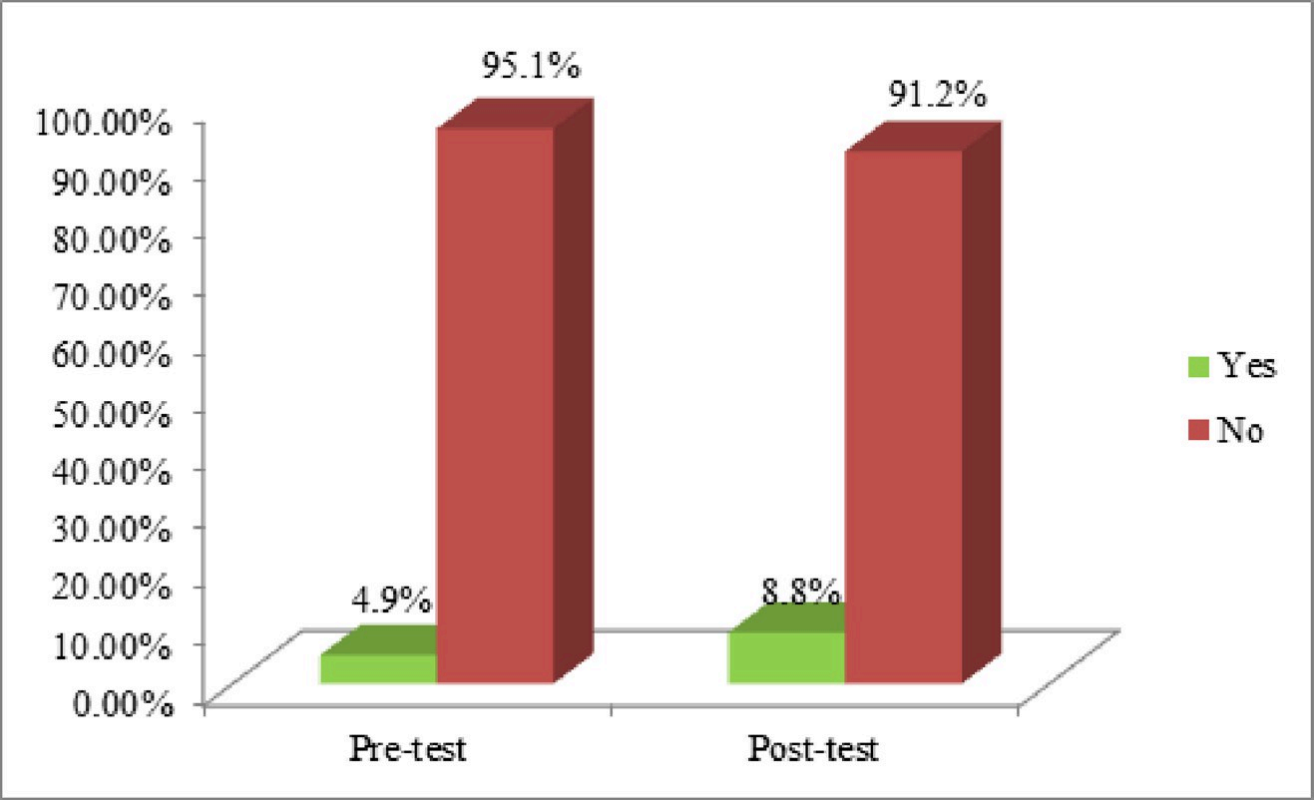
Comparison between pre-and post-test of participants based on the practice level of Pap test (n = 102).

Before mHealth intervention 5 (4.9%) of them had got their Pap test, whereas after intervention 9 (8.8%) of them had got their Pap test (**Fig 2**).

The difference between the median knowledge scores regarding warning signs and symptoms, risk factors, HPV vaccination and practice score for the Pap test was assessed using the Wilcoxon Sign Rank test (**Table 3**).

**Table 3:** Comparison of knowledge median scores regarding warning signs & symptoms, risk factors, and HPV vaccination and practice scores of Pap test before and after the intervention (n = 102).

Variables	Pre-test (IQR)	Post-test (IQR)	p-value
**Knowledge Scores regarding warning signs & symptoms**	0	14 (13–18)	**<0.001**
**Knowledge scores regarding risk factors**	33 (33)	37 (31–41)	**<0.001**
**Knowledge scores regarding HPV Vaccination**	1 (1–2)	3 (2–3)	**<0.001**
**Practice scores regarding Pap test**	1 (1)	1 (1)	0.102

IQR- Inter-quartile range

Kruskal Wallis test was done to assess the difference between median knowledge and practice scores across age categories, education category, socioeconomic status, and marital status before mHealth intervention. Mann Whitney U test was done to assess the difference between median knowledge and practice scores across occupational status before mHealth intervention. It was found that the difference between median knowledge scores of warning signs & symptoms across occupational status was statistically significant (**Table 4**), meaning there was not much difference between the median scores across socio-demographic variables except for employed women in the knowledge scores of warning signs & symptoms.

**Table 4:** Comparison between median knowledge scores of warning signs and symptoms, risk factors of cervical cancer and HPV vaccination and median practice scores of Pap test across socio-demographic variables before mHealth intervention (n = 102).

Socio-demographic variables	Knowledge scores of warning signs & symptoms		Knowledge scores of risk factors		Knowledge scores of HPV vaccination		Practice scores of Pap test	
	Median (IQR)	p-value	Median (IQR)	p-value	Median (IQR)	p-value	Median (IQR)	p-value
**Age Category (in years)**								
18–30	0	0.08	33	0.432	1 (1–2)	0.927	2	0.413
31–45	0		33		1 (1–2)		2	
46–60	0		33		1 (1–2)		2	
**Education Category**								
Illiterate	0	0.06	33	0.851	1 (1–2)	0.624	2	0.492
Schooling	0		33		1 (1–2)		2	
Beyond 10 years of schooling	0		33		1 (1–2)		2	
**Occupation status**	00 (0–6)	**<0.05**		0.459		0.941		0.083
Unemployed			3333		1 (1–2)1		22	
Employed					(1–2)			
**Socio-economic status**								
Lower middle	0	0.929	33	0.459	1 (1–2)	0.706	2	0.279
Upper lower	0		33		1 (1–2)		2	
Lower	0 (0–3)		33 (32–33)		1 (1–2)		2	
**Marital status**								
Single	0 (0–2)	0.703	33 (33–34)	0.291	1	0.191	2	0.777
Married	0		33		1 (1–2)		2	
Widow	0		33		1 (1–1.75)		2	

IQR- Inter-quartile range

Kruskal Wallis test was done to assess the difference between median knowledge and practice scores across age categories, education category, socioeconomic status, and marital status after mHealth intervention. Mann Whitney U test was done to assess the difference between median knowledge and practice scores across occupational status after mHealth intervention. It was found that the difference between median knowledge scores of warning signs & symptoms, risk factors, and median practice scores of Pap test across occupational status was statistically significant (**Table 5**). And also, the difference between median knowledge scores of warning signs & symptoms, and risk factors across marital status was statistically significant (**Table 5**). There was a significant increase in the knowledge about warning signs and symptoms and risk factors among the employed and single women.

**Table 5:** Comparison between median knowledge scores of warning signs and symptoms, risk factors of cervical cancer and HPV vaccination and median practice scores of Pap test across socio-demographic variables after mHealth intervention (n = 102).

Socio-demographic variables	Knowledge scores of warning signs & symptoms		Knowledge scores of risk factors		Knowledge scores of HPV vaccination		Practice scores of Pap test	
	Median (IQR)	p-value	Median (IQR)	p-value	Median (IQR)	p-value	Median (IQR)	p-value
**Age Category (in years)**								
18–30	15 (14–18)		38 (32–41)		3 (2–3)		2	
31–45	15 (13–17)	0.279	38 (32–42)	0.528	3 (2–3)	0.749	2	0.103
46–60	14 (10.25–17)		35.5 (29.25–41)		3 (2–3)		2	
**Education Category**								
Illiterate	14 (10–17)	0.242	35 (29–39)	0.122	3 (2–3)	0.251	2	0.637
Schooling	15 (15–18)		38 (31–42)		3 (2–3)		2	
Beyond 10 years of schooling	14 (14–18)		37 (33–40.5)		3 (2–3)		2	
**Occupation status**								
Unemployed	14 (13–16)	**<0.05**	35 (31–40)	**<0.05**	3 (2–3)	0.636	2	**<0.05**
Employed	17 (14–20)		39 (35–42)		3 (2–3)		2	
**Socio-economic status**								
Lower middle	14 (13–18)	0.965	37 (32–41)	0.764	3 (2–3)	0.797	2	0.112
Upper lower	15 (13–18)		37 (31–41)		3 (2–3)		2	
Lower	14.5 (12–21)		36 (23.75–40.5)		3 (2–3)		2	
**Marital status**								
Single	21 (16–21)	**<0.05**	44 (38–46.5)	**<0.05**	3 (2.5–3)	0.195	2	0.623
Married	14 (14–17.5)		37 (31–41)		3 (2–3)		2	
Widow	10.5 (9.25–13.25)		36 (30.75–40.5)		2 (2–2.75)		2	

IQR- Inter-quartile range

## Discussion

The prevalence of cancer is increasing globally, and so will cancer mortality without intervention [20]. Dr. Sankaranarayanan states that "multiple diseases need various forms of action [under the cancer label]." Some are primarily susceptible to prevention; some need intensive rehabilitation; some need early diagnosis and therapy. The need for a multipronged solution in the form of cancer preparedness is widely recognized. Such a plan incorporates intervention in a wide variety of areas, including prevention, early diagnosis, recovery, advocacy for survivors, and palliative care [20]. Like other lower- and middle-income countries, India should concentrate its attention on closing the chronic access gaps and ensuring the quality of care across the cancer spectrum through enhanced facilities and service supply, while prioritizing healthier lifestyle promotion [21].

In the current study, cancer preparedness was assessed by evaluating the level of awareness regarding warning signs and symptoms, risk factors of cervical cancer, awareness of HPV vaccination; and practice level concerning the Pap test. Health education intervention was taken up to improve cancer preparedness.

Before the intervention, only one-eighth of the study participant had heard about cervical cancer, and overall had very low knowledge about warning signs and symptoms, risk factors of cervical cancer, and only around 5% of study subjects had got their Pap test. A study done in Greece by Vasiliki Dalla et al, using a cervical cancer awareness measure tool had low awareness about both warning signs and risk factors of cervical cancer which is similar to the current study [22]. A study done among HPV-positive women in Yunnan province showed that only half of them knew about cervical cancer [23]. A Ugandan study revealed that almost everyone (99%) knew about cervical cancer signs, symptoms, and risk factors, because they got their information regularly, which was not observed in the present study [24]. High awareness about cervical cancer, its signs, symptoms, and risk factors provides firm anchorage for targeted interventions to increase uptake of preventive methods and promote early health‐seeking for cervical cancer symptoms [24]. Hence there is a need for health education in the present study population.

Health education is a crucial strategy in the development of health-promoting behavior; its serious implications are promoting health, prevention of the diseases, and their management [25]. Information and communication technologies and digital devices such as smartphones, provide a potentially powerful means of patient education and strengthening behavioral change [26]. According to India’s Family Health Survey 2020, not only is the number of women who possess mobile phones continuously growing, but more women than ever before are utilising the device individually rather than depending on family members for mobile access [27]. The use of smartphones is transforming the provision of patient education in health care through the realistic, personalized, and contextually relevant delivery of information and treatments [28]. The use of audio or visual formats provides additional means of communication for the transmission of educational information that may be difficult to communicate through words alone [29]. Besides, audio or visual educational aids can improve the patient’s comprehension of a particular condition or procedure [29]. In this study, we used mHealth health education application in the form of interactive voice response to educate the women in social support groups. After the intervention, there was a significant eighteen times increase in the knowledge about warning signs and symptoms, around twenty times increase in the knowledge about risk factors of cervical cancer, and around thirty times increase in knowledge about HPV vaccination. Although there was a slight increase (around 5%) in women getting their Pap test it was not statistically significant. As per our knowledge, there are not many studies done to assess the improvement in knowledge about cervical cancer after implementing mHealth intervention.

A study done in Africa by Akaninyene Otu et al used mHealth intervention in improving the knowledge about Ebola among frontline health workers. There was a modest improvement in the knowledge of the health workers which indicated that mHealth applications may hold promise for training health workers and developing resilient health systems to respond to epidemics [30]. In another study on opioid use disorder subjects, mHealth was used to improve the knowledge about HIV and Hepatitis C virus, and there was a significant increase in their knowledge after the implementation of the intervention [31].

A voice-based mHealth intervention was used in Mumbai to improve the Infant Care knowledge, and practices among low-income women. It significantly improved the infant care practices and maternal knowledge that can positively impact infant child health like ideal body weight of the infant, and when to start the supplementary feeding [32]. With the advancement in time, it has proven that mHealth has helped in improving the knowledge, but it requires reinforcement and revisions. These studies have concluded that a time- and cost-effective solution can be delivered through mobile health education interventions.

Social support groups were involved in the current study so that the information could disseminate to the general population. Sintayehu Hailu Alemu et al, in their study which dealt with the impact of social support groups in apple production on empowering women in Ethiopia, findings pointed towards the optimistic and important influence of social support group engagement on community-level equality, which indicates that social support groups provide women with efficient space to exchange knowledge [33]. Similarly, a study done by Kalaiah Fleming et al, proved that using social support group women to offer knowledge and tools on cervical cancer/HPV screening tends to influence other women. Further, it could be a favorable and practical educational strategy to increase and extend access and availability to the at-risk population of culturally appropriate information and services [5].

### Strength

The study focused on using technology to empower the women regarding cervical cancer and involved both working and non-working women in social support groups, in a view to disseminate the information to the community faster.

### Limitation

Initial plan of the study was to do a comparison between low, middle and high socio-economic status women groups. Due to the COVID situation, middle- and higher-socioeconomic status women were not able to take part in the study and the sample was restricted to 102 women.

## Conclusion

We conclude that cervical cancer preparedness through mHealth educational intervention saw an increase in the knowledge about warning signs and symptoms, risk factors of cervical cancer, and knowledge about HPV vaccination, and there was a slight increase (around 5%) in women getting their Pap test. A follow-up study is required to check whether the participants got their Pap test done or not, after receiving the reminder message to get the Pap test done and to check whether the information about cervical cancer got disseminated to the general population or not.

## Supporting information

S1 File Annexures. (DOCX)

S2 File m Health application pictures. (ZIP)
