# Supplementary material for: Effects of mobile Health (mHealth) application on cervical cancer prevention knowledge and screening among women social support groups with low-socioeconomic status in Mysuru city, Southern India
Source: PLoS One. 2022 Sep 1;17(9):e0273070. doi: 10.1371/journal.pone.0273070 (PMC9436151; doi:10.1371/journal.pone.0273070)
Supplement: S1 File — (DOCX) [file pone.0273070.s001.docx]

***Annexure***

**JSS ACADEMY OF HIGHER EDUCATION AND RESEARCH**

**JSS MEDICAL COLLEGE, MYSURU-15**

**DEPARTMENT OF COMMUNITY MEDICINE**

**INFORMATION SHEET**

**TITLE OF THE STUDY:** EFFECTS OF MOBILE HEALTH (mHealth) APPLICATION ON CERVICAL CANCER PREVENTION KNOWLEDGE AND SCREENING AMONG WOMEN SOCIAL SUPPORT GROUPS WITH LOW-SOCIOECONOMIC STATUS IN MYSURU CITY, SOUTHERN INDIA

**Introduction**

Cervical cancer is the cancer which is formed in the tissues of cervix; it is a slow growing tumor that might not have any symptoms at all. There are different methods for screening cervical cancer, which are cost-effective but people do not attend regular screening. Mobile Health (m-Health) technologies will help us to promote health care by providing information about health care, so that it can improve the access to health care services for all. This study intends to empower women among social support groups about cervical cancer preparedness using mobile health (m-health) applications.

**Methodology**

- Information regarding your age, gender, education, occupation, income, marital status, parity will be obtained using a proforma.
- Knowledge about cervical cancer will be assessed by a questionnaire.
- Mobile application will be developed after taking inputs from women.
- At the end, end line assessment will be done using post-test questionnaire and focus group discussions.
- Confidentiality will be maintained and the findings will be used for research purposes only.
- All financial costs will be taken by the researcher.

**For any queries kindly Contact**

**Researcher: Guide:**

Dr. Chandana H Dr Madhu B

(9535252290) (9480134678)

**JSS ACADEMY OF HIGHER EDUCATION AND RESEARCH**

**JSS MEDICAL COLLEGE, MYSURU – 15**

**DEPARTMENT OF COMMUNITY MEDICINE**

**INFORMED CONSENT FORM**

Subject’s Initials: _______________

Subject’s Name: _____________________________

Date of Birth / Age: _________________

I have been explained in detail in my own understandable language about the purpose of the study that is to empower the women social support groups about cervical cancer preparedness using m-health application. This study will help in increasing the knowledge about cervical cancer and will increase willingness and adherence to screening. I am willingly giving my consent to participate in this study. I have been informed that my name and medical details will be kept confidential.

|  |  | Please initial box  (Subject) | |
| --- | --- | --- | --- |
| (i) | I confirm that I have read and understood the information sheet dated ___ for the above study and have had the opportunity to ask questions. | [         ] | |
| (ii) | I understand that my participation in the study is voluntary and that I am free to withdraw at any time, without giving any reason, without my medical care or legal rights being affected. | [         ] | |
| (iii) | I understand that the Ethics Committee and the regulatory authorities will not need my permission to look at my health records both in respect of the current study and any further research that may be conducted in relation to it, even if I withdraw from the trial.  However, I understand that my identity will not be revealed in any information released to third parties or published. | [         ] | |
| (iv) | I agree not to restrict the use of any data or results that arise from this study provided such a use is only for scientific purpose(s) | [        ] | |
| (v) | I agree to take part in the above study. | [         ] | |
|  |  |  |  |

Signature (or Thumb impression) of the Subject with date: _____________

Signature of the Witness

______________________               Date: _____/_____/_______

Name of the Witness:

_____________________________

Name and Signature of the Principal Investigator with date: ________________________________________________

**PRE- &POST-TEST QUESTIONNAIRE IN mHEALTH**

1. Name:
2. Age:
3. Address:

1. Mobile No.:
2. Education: Illiterate/Primary school/Middle School/ High school/ Intermediate or diploma/Graduate/ Post graduate
3. Occupation: Unemployed/ elementary occupation/ plant and machine operators/ Craft and related trade workers/ Skilled agriculture workers/ Shop and market sales workers/ Clerks/ Technicians/ Associate professionals/ Professionals/ Legislators, senior officials & managers
4. Per capita income:
5. Marital status: Single/never married/married/separated/divorced/widowed
6. Have you, your family or close friends had cancer?

Yes No Don’t know Prefer not to say

You □ □ □ □

Close family member □ □ □ □

Oher family member □ □ □ □

Close friend □ □ □ □

Other friend □ □ □ □

1. The following may or may not be warning signs for cervical cancer. We are interested in your opinion:

Yes No Don’t know

1. Do you think vaginal bleeding between periods □ □ □

Could be a sign of cervical cancer?

1. Do you think persistent low back pain could □ □ □

be a sign of cervical cancer?

1. Do you think a persistent vaginal discharge □ □ □

That smells unpleasant could be a sign of cervical

Cancer?

1. Do you think discomfort or pain during sex could □ □ □

Be a sign of cervical cancer?

1. Do you think menstrual periods that are heavier or □ □ □

Longer than usual could be a sign of cervical

Cancer?

1. Do you think persistent diarrhea could be a sign □ □ □

Of cervical cancer?

1. Do you think vaginal bleeding after the menopause □ □ □

Could be a sign of cervical cancer?

1. Do you think persistent pelvic pain could be a □ □ □

Sign of cervical cancer?

1. Do you think vaginal bleeding during or after sex □ □ □

Could be a sign of cervical cancer?

1. Do you think blood in urine or stool could be a □ □ □

Sign of cervical cancer?

1. Do you think unexplained weight loss could be □ □ □

A sign of cervical cancer?

1. In the next year, who is most likely to develop cervical cancer?^6^
   1. A woman aged 20 to 29 years
   2. A woman aged 30 to 49 years
   3. A woman aged 50 to 69 years
   4. A woman aged 70 or over
   5. Cervical cancer is unrelated to age
2. The following may or may not increase a woman’s chance of developing cervical cancer. How much do you agree that each of these can increase a woman’s chance of developing cervical cancer?

| Strongly | Disagree | Not sure | Agree | Strongly |
| --- | --- | --- | --- | --- |
| disagree |  |  |  | agree |
|  |  |  |  |  |

Infection with HPV (human □ □ □ □ □

papillomavirus)

Smoking any cigarettes at all □ □ □ □ □

Having a weakened immune system

(e.g. because of HIV/AIDS, □ □ □ □ □

immunosuppressant drugs or having

a transplant)

Long term use of the contraceptive □ □ □ □ □

pill

Infection with Chlamydia (a sexually □ □ □ □ □

transmitted infection)

Having a sexual partner who is not □ □ □ □

circumcised

Starting to have sex at a young age □ □ □ □

(before age 17)

=

Having many sexual partners □ □ □ □

Having many children □ □ □ □

Having a sexual partner with many □ □ □ □

previous partners

Not going for regular smear (Pap) tests □ □ □ □

1. How confident are you that you would notice a cervical cancer symptom?

Not at all confident Not very confident Fairly confident Very confident

□ □ □ □

1. Have you had a pap test before in your life? Yes/No/Prefer not to say
2. As far as you are aware, is there a vaccination to protect against cervical cancer?

Yes □ No □ Don’t know □
