# Supplementary figures and images for: Effects of mobile Health (mHealth) application on cervical cancer prevention knowledge and screening among women social support groups with low-socioeconomic status in Mysuru city, Southern India
Source: PLoS One. 2022 Sep 1;17(9):e0273070. doi: 10.1371/journal.pone.0273070 (PMC9436151; doi:10.1371/journal.pone.0273070)

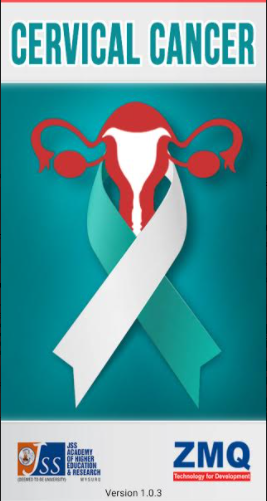

Supplement: S2 File — (ZIP) [file pone.0273070.s002.zip › 1.PNG]

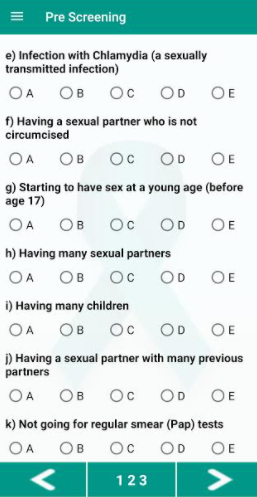

Supplement: S2 File — (ZIP) [file pone.0273070.s002.zip › 10.PNG]

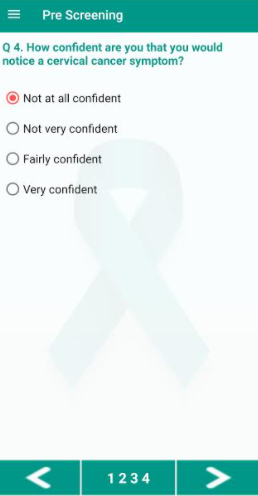

Supplement: S2 File — (ZIP) [file pone.0273070.s002.zip › 11.PNG]

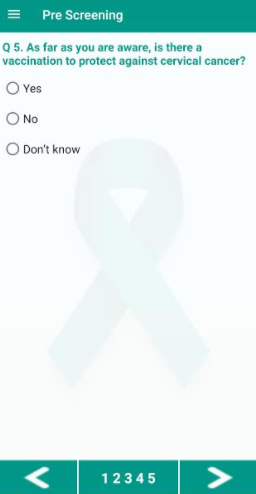

Supplement: S2 File — (ZIP) [file pone.0273070.s002.zip › 12.PNG]

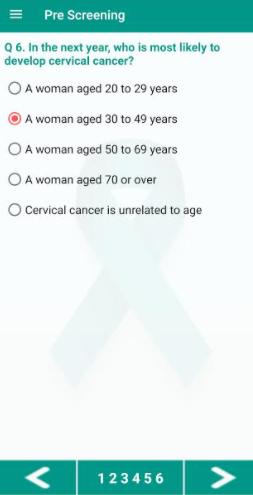

Supplement: S2 File — (ZIP) [file pone.0273070.s002.zip › 13.PNG]

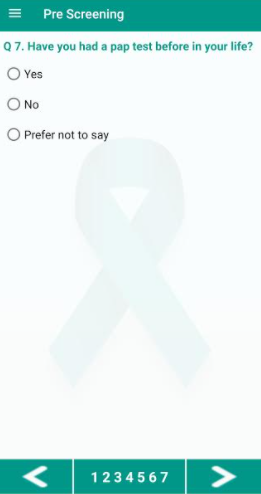

Supplement: S2 File — (ZIP) [file pone.0273070.s002.zip › 14.PNG]

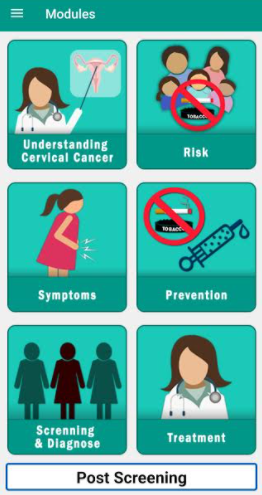

Supplement: S2 File — (ZIP) [file pone.0273070.s002.zip › 15.PNG]

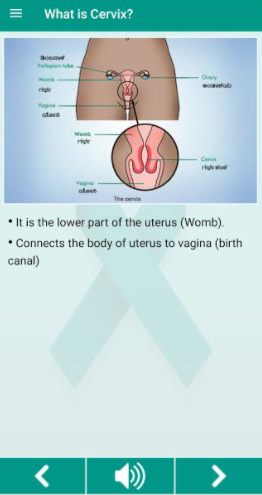

Supplement: S2 File — (ZIP) [file pone.0273070.s002.zip › 16.PNG]

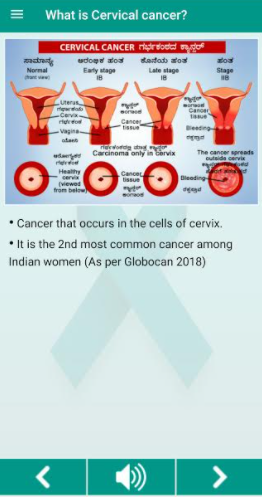

Supplement: S2 File — (ZIP) [file pone.0273070.s002.zip › 17.PNG]

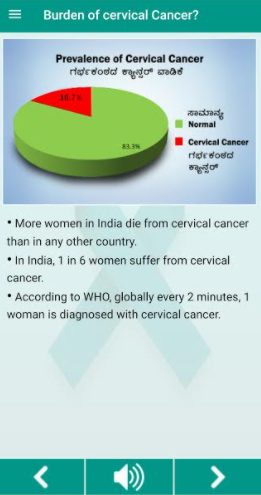

Supplement: S2 File — (ZIP) [file pone.0273070.s002.zip › 18.PNG]

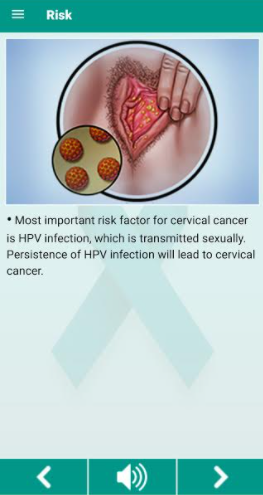

Supplement: S2 File — (ZIP) [file pone.0273070.s002.zip › 19.PNG]

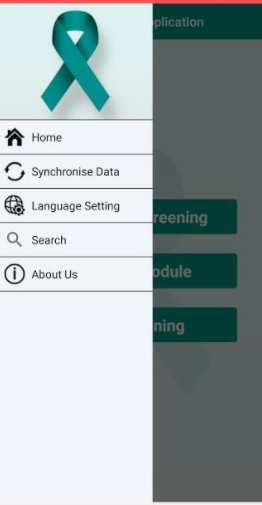

Supplement: S2 File — (ZIP) [file pone.0273070.s002.zip › 2.PNG]

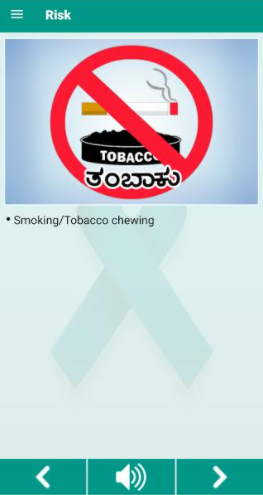

Supplement: S2 File — (ZIP) [file pone.0273070.s002.zip › 20.PNG]

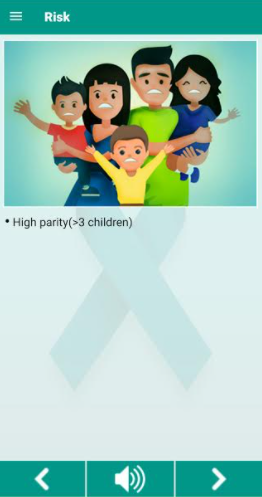

Supplement: S2 File — (ZIP) [file pone.0273070.s002.zip › 21.PNG]

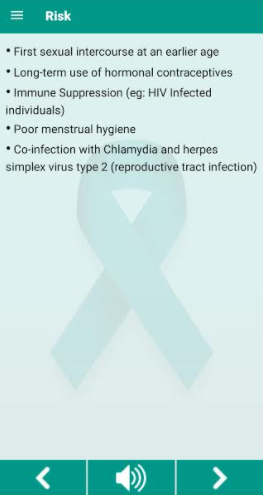

Supplement: S2 File — (ZIP) [file pone.0273070.s002.zip › 22.PNG]

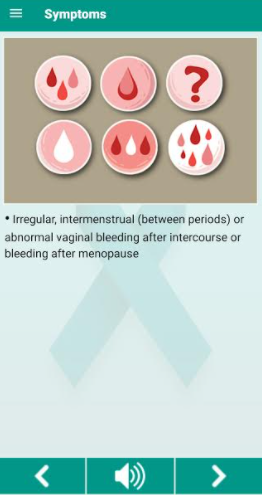

Supplement: S2 File — (ZIP) [file pone.0273070.s002.zip › 23.PNG]

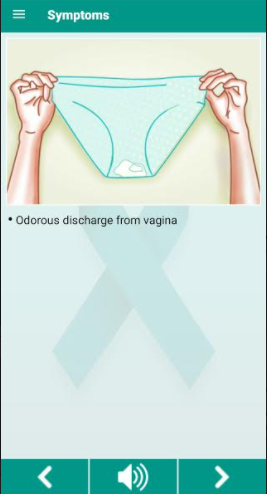

Supplement: S2 File — (ZIP) [file pone.0273070.s002.zip › 24.PNG]

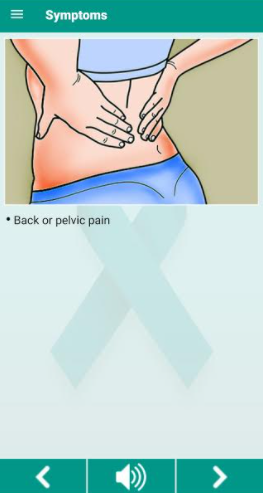

Supplement: S2 File — (ZIP) [file pone.0273070.s002.zip › 25.PNG]

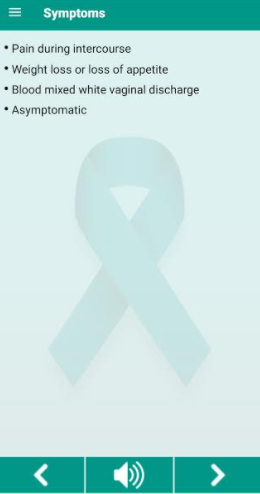

Supplement: S2 File — (ZIP) [file pone.0273070.s002.zip › 26.PNG]

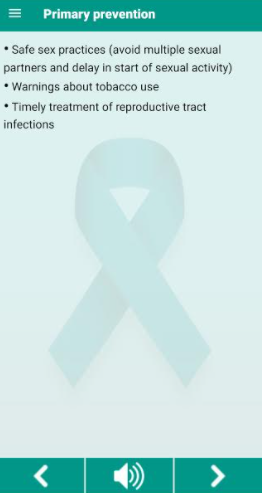

Supplement: S2 File — (ZIP) [file pone.0273070.s002.zip › 27.PNG]

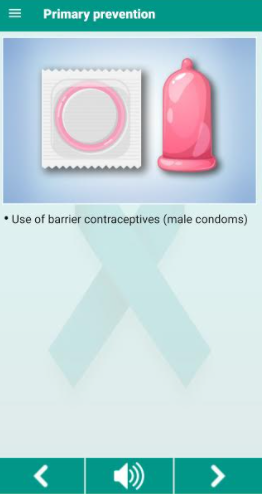

Supplement: S2 File — (ZIP) [file pone.0273070.s002.zip › 28.PNG]

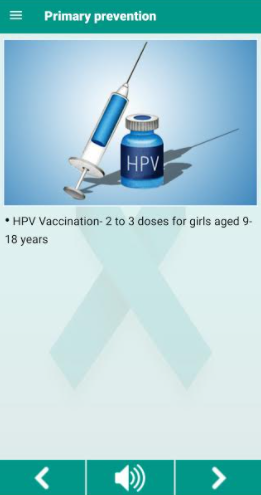

Supplement: S2 File — (ZIP) [file pone.0273070.s002.zip › 29.PNG]

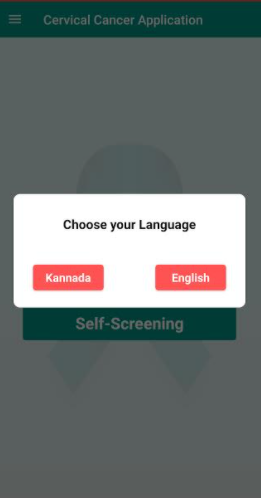

Supplement: S2 File — (ZIP) [file pone.0273070.s002.zip › 3.PNG]

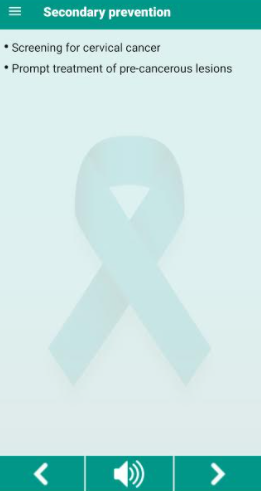

Supplement: S2 File — (ZIP) [file pone.0273070.s002.zip › 30.PNG]

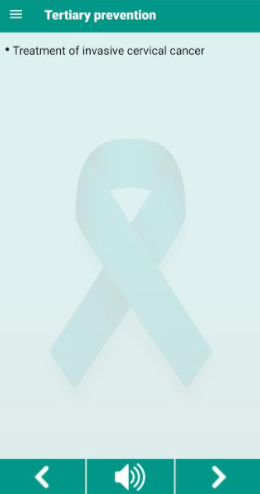

Supplement: S2 File — (ZIP) [file pone.0273070.s002.zip › 31.PNG]

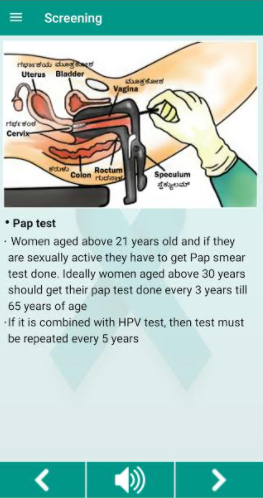

Supplement: S2 File — (ZIP) [file pone.0273070.s002.zip › 32.PNG]

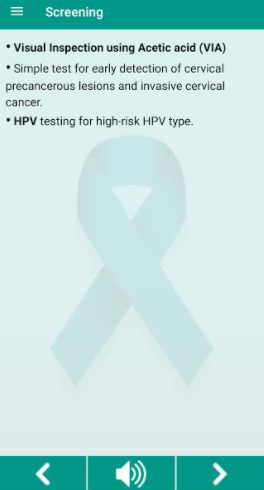

Supplement: S2 File — (ZIP) [file pone.0273070.s002.zip › 33.PNG]

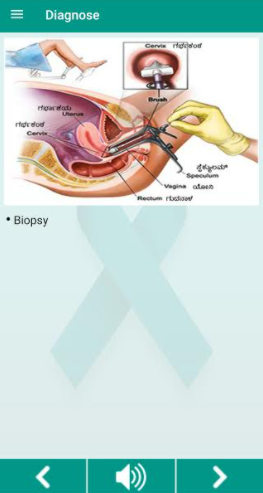

Supplement: S2 File — (ZIP) [file pone.0273070.s002.zip › 34.PNG]

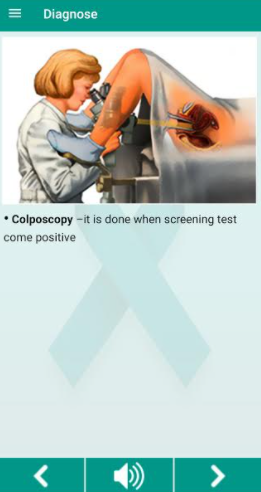

Supplement: S2 File — (ZIP) [file pone.0273070.s002.zip › 35.PNG]

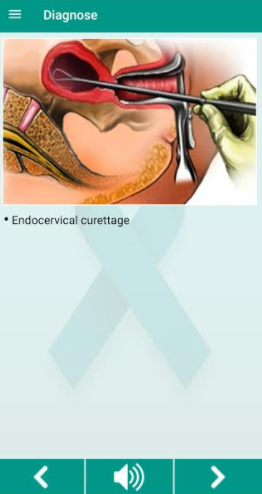

Supplement: S2 File — (ZIP) [file pone.0273070.s002.zip › 36.PNG]

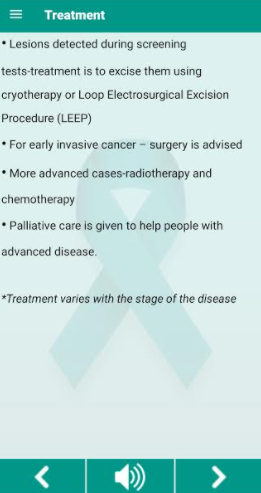

Supplement: S2 File — (ZIP) [file pone.0273070.s002.zip › 37.PNG]

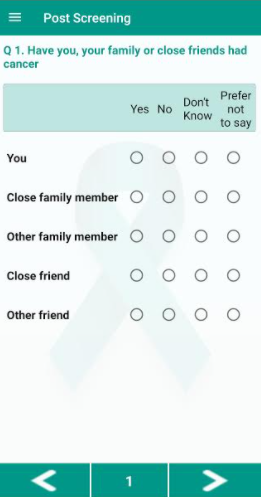

Supplement: S2 File — (ZIP) [file pone.0273070.s002.zip › 38.PNG]

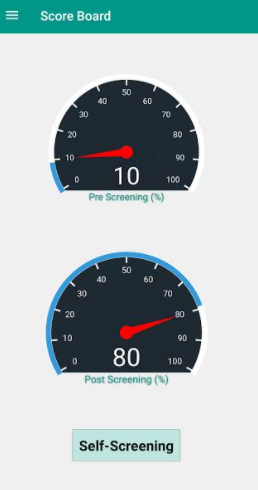

Supplement: S2 File — (ZIP) [file pone.0273070.s002.zip › 39.PNG]

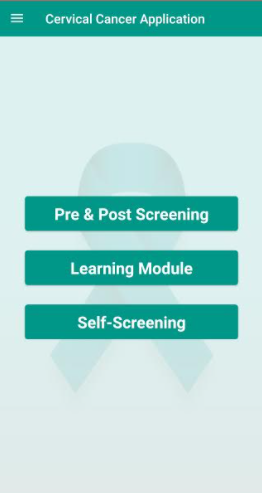

Supplement: S2 File — (ZIP) [file pone.0273070.s002.zip › 4.PNG]

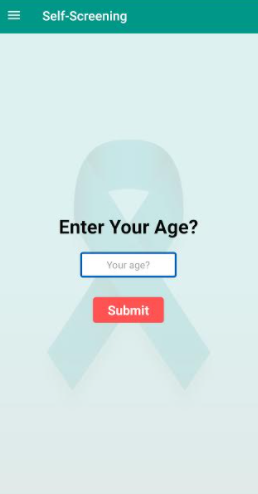

Supplement: S2 File — (ZIP) [file pone.0273070.s002.zip › 40.PNG]

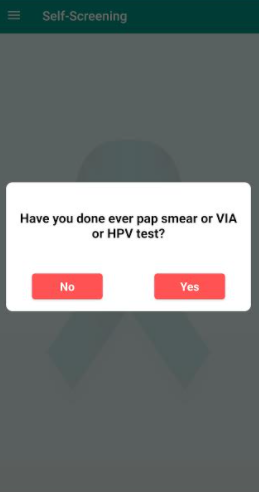

Supplement: S2 File — (ZIP) [file pone.0273070.s002.zip › 41.PNG]

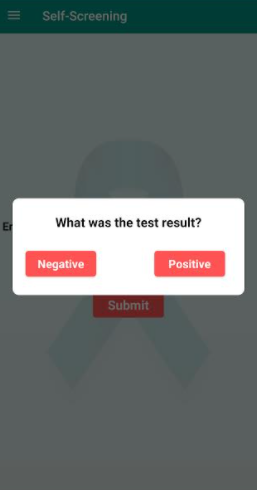

Supplement: S2 File — (ZIP) [file pone.0273070.s002.zip › 42.PNG]

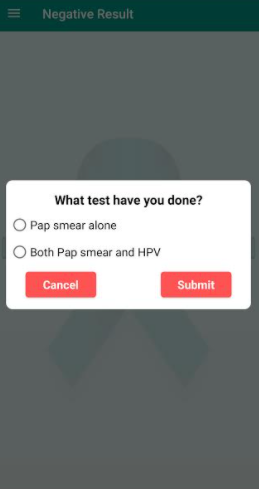

Supplement: S2 File — (ZIP) [file pone.0273070.s002.zip › 43.PNG]

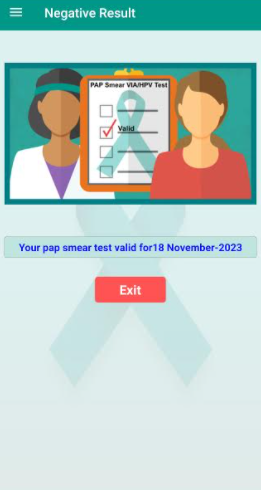

Supplement: S2 File — (ZIP) [file pone.0273070.s002.zip › 44.PNG]

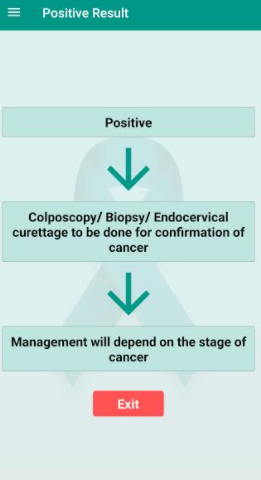

Supplement: S2 File — (ZIP) [file pone.0273070.s002.zip › 45.PNG]

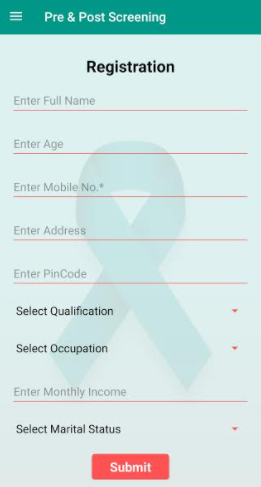

Supplement: S2 File — (ZIP) [file pone.0273070.s002.zip › 5.PNG]

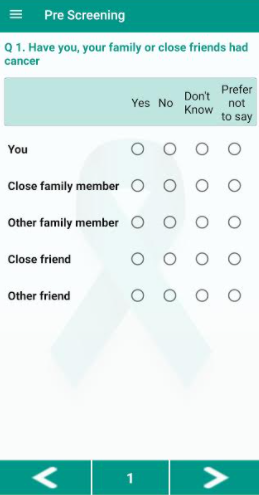

Supplement: S2 File — (ZIP) [file pone.0273070.s002.zip › 6.PNG]

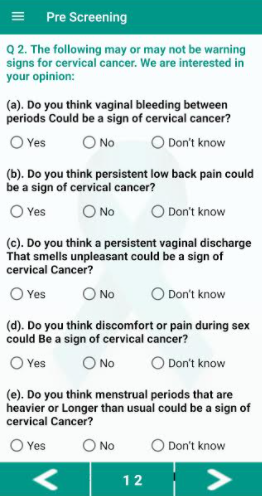

Supplement: S2 File — (ZIP) [file pone.0273070.s002.zip › 7.PNG]

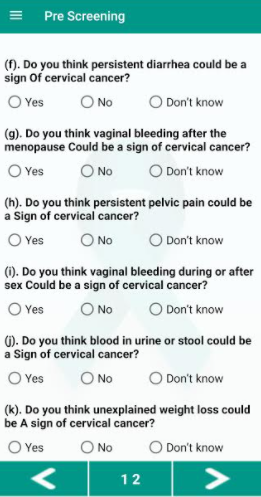

Supplement: S2 File — (ZIP) [file pone.0273070.s002.zip › 8.PNG]

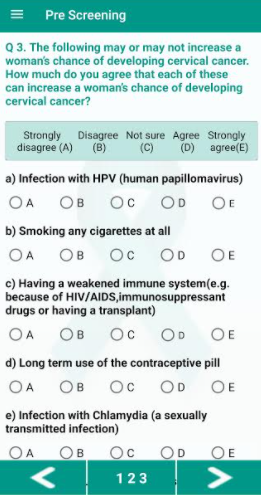

Supplement: S2 File — (ZIP) [file pone.0273070.s002.zip › 9.PNG]
